# Supplementary material for: Crawling and Gliding: A Computational Model for Shape-Driven Cell Migration
Source: PLoS Comput Biol. 2015 Oct 21;11(10):e1004280. doi: 10.1371/journal.pcbi.1004280 (PMC4619082; doi:10.1371/journal.pcbi.1004280)
Supplement: S1 Code — (ZIP) [file pcbi.1004280.s012.zip › release/tst/doc/html/parameter_8h_source.html]

Tissue Simulation Toolkit: parameter.h Source File


Tissue Simulation Toolkit
 0.1.4.1


- Main Page
- Namespaces
- Classes
- Files

- File List
- File Members

parameter.h

Go to the documentation of this file.

1 /\*

2

3 Copyright 1996-2006 Roeland Merks

4

5 This file is part of Tissue Simulation Toolkit.

6

7 Tissue Simulation Toolkit is free software; you can redistribute

8 it and/or modify it under the terms of the GNU General Public

9 License as published by the Free Software Foundation; either

10 version 2 of the License, or (at your option) any later version.

11

12 Tissue Simulation Toolkit is distributed in the hope that it will

13 be useful, but WITHOUT ANY WARRANTY; without even the implied

14 warranty of MERCHANTABILITY or FITNESS FOR A PARTICULAR PURPOSE.

15 See the GNU General Public License for more details.

16

17 You should have received a copy of the GNU General Public License

18 along with Tissue Simulation Toolkit; if not, write to the Free

19 Software Foundation, Inc., 51 Franklin St, Fifth Floor, Boston, MA

20 02110-1301 USA

21

22 \*/

23 #ifndef \_PARAMETER\_H\_

24 #define \_PARAMETER\_H\_

25

26 #include <iostream>

27 using namespace std;

28 class Parameter {

29

30  public:

31  Parameter();

32  ~Parameter();

33  void CleanUp(void);

34  void Read(const char \*filename);

35  void Write(ostream &os) const;

36  double T;

37  int target\_area;

38  int target\_length;

39  double lambda;

40  double lambda2;

41  char \* Jtable;

42  int conn\_diss;

43  bool vecadherinknockout;

44  bool extensiononly;

45  int chemotaxis;

46  int border\_energy;

47  int neighbours;

48  bool periodic\_boundaries;

49  int n\_chem;

50  double \* diff\_coeff;

51  double \* decay\_rate;

52  double \* secr\_rate;

53  double saturation;

54  double dt;

55  double dx;

56  int pde\_its;

57  int n\_init\_cells;

58  int size\_init\_cells;

59  int sizex;

60  int sizey;

61  int divisions;

62  int mcs;

63  int rseed;

64  double subfield;

65  int relaxation;

66  int storage\_stride;

67  bool graphics;

68  bool store;

69  char \* datadir;

70  private:

71 };

72

73 ostream &operator<<(ostream &os, Parameter &p);

74 const char \*sbool(const bool &p);

75

76

77 #endif

Parameter::periodic\_boundaries

bool periodic\_boundaries

**Definition:** parameter.h:48

Parameter::chemotaxis

int chemotaxis

**Definition:** parameter.h:45

Parameter::T

double T

**Definition:** parameter.h:36

Parameter::extensiononly

bool extensiononly

**Definition:** parameter.h:44

Parameter::vecadherinknockout

bool vecadherinknockout

**Definition:** parameter.h:43

Parameter::relaxation

int relaxation

**Definition:** parameter.h:65

Parameter::sizey

int sizey

**Definition:** parameter.h:60

Parameter::pde\_its

int pde\_its

**Definition:** parameter.h:56

Parameter::divisions

int divisions

**Definition:** parameter.h:61

Parameter::storage\_stride

int storage\_stride

**Definition:** parameter.h:66

Parameter::neighbours

int neighbours

**Definition:** parameter.h:47

Parameter::store

bool store

**Definition:** parameter.h:68

operator<<

ostream & operator<<(ostream &os, Parameter &p)

**Definition:** parameter.cpp:205

Parameter::target\_area

int target\_area

**Definition:** parameter.h:37

Parameter::subfield

double subfield

**Definition:** parameter.h:64

Parameter::n\_chem

int n\_chem

**Definition:** parameter.h:49

Parameter::target\_length

int target\_length

**Definition:** parameter.h:38

Parameter::dt

double dt

**Definition:** parameter.h:54

Parameter::n\_init\_cells

int n\_init\_cells

**Definition:** parameter.h:57

Parameter::sizex

int sizex

**Definition:** parameter.h:59

Parameter

**Definition:** parameter.h:28

Parameter::rseed

int rseed

**Definition:** parameter.h:63

Parameter::saturation

double saturation

**Definition:** parameter.h:53

Parameter::datadir

char \* datadir

**Definition:** parameter.h:69

Parameter::size\_init\_cells

int size\_init\_cells

**Definition:** parameter.h:58

sbool

const char \* sbool(const bool &p)

**Definition:** parameter.cpp:151

Parameter::diff\_coeff

double \* diff\_coeff

**Definition:** parameter.h:50

Parameter::secr\_rate

double \* secr\_rate

**Definition:** parameter.h:52

Parameter::Jtable

char \* Jtable

**Definition:** parameter.h:41

Parameter::conn\_diss

int conn\_diss

**Definition:** parameter.h:42

Parameter::lambda

double lambda

**Definition:** parameter.h:39

Parameter::decay\_rate

double \* decay\_rate

**Definition:** parameter.h:51

Parameter::mcs

int mcs

**Definition:** parameter.h:62

Parameter::lambda2

double lambda2

**Definition:** parameter.h:40

Parameter::border\_energy

int border\_energy

**Definition:** parameter.h:46

Parameter::dx

double dx

**Definition:** parameter.h:55

Parameter::graphics

bool graphics

**Definition:** parameter.h:67


---

Generated on Thu Aug 14 2014 22:04:01 for Tissue Simulation Toolkit by  

 1.8.6
